# Gaming Green: The Educational Potential of Eco – A Digital Simulated Ecosystem

**DOI:** 10.3389/fpsyg.2019.02846

**Published:** 2019-12-19

**Authors:** Kristoffer S. Fjællingsdal, Christian A. Klöckner

**Affiliations:** Department of Psychology, Norwegian University of Science and Technology, Trondheim, Norway

**Keywords:** serious games, sustainability, ecosystems, environmental consciousness, environmental media

## Abstract

Research into the use of videogames in education is on the rise, and they are cementing their position as part of the modernized, digital classroom. Sustainability education has also become a subject of interest among environmentally minded game developers and understanding the educational impact of such games is rapidly becoming an important field. This study examined the educational potential of the digital simulated ecosystem known as Eco, in order to reveal how playing Eco might promote environmental consciousness surrounding ecosystems. Qualitative data from seven respondents were subjected to a thematic analysis, revealing two main themes that highlight both game-based learning outcomes as well as barriers against learning. The findings indicate that Eco is a viable tool for promoting some aspects of environmental consciousness about ecosystems, and suggestions for future implementation of Eco are provided.

## Introduction

Videogames represent one of the fastest growing media trends, with an estimation of 2.5 billion people playing them globally (WEPC, 2018). Aside from their use in entertainment (Sweetser and Wyeth, 2005), videogames are also used in education as so-called *serious games* (Wouters et al., 2013). For decades, researchers have shown interest in utilizing such games to educate the public about *sustainability issues* (e.g., Sandbrook et al., 2015; Waddington and Fennewald, 2018).

There is a strong scientific consensus that anthropogenic climate change is occurring (Cook et al., 2013), and that it causes a wide array of negative alterations in oceanic life (Lejeusne et al., 2009), plant disease rates (Garrett et al., 2006), and biodiversity conservation issues (Salafsky et al., 2002; Redpath et al., 2018). Environmental education about these issues can steer human behavior toward a more harmonious relationship with nature (Varela-Candamio et al., 2018). In order to educate the public about environmental issues, novel and creative methodologies are required (Klöckner, 2015). One way of communicating environmental issues is through videogames, due to their long history of raising awareness, educating and presenting contemporary research (Eisenack and Reckien, 2013).

A new addition to the library of games focusing on the environment is *Eco*. It is a *simulated ecosystem* where players must collaborate and build technology to destroy a meteor rushing toward the Earth, while simultaneously preventing harmful pollutants from escaping into the surrounding nature (Strange Loop Games, 2018a). Drawing on interdisciplinary theoretical insight from fields such as psychology, game theory and sustainability, this article examines how playing Eco might promote environmental consciousness surrounding ecosystems.

### Eco – What Is It, and How Does It Work?

Eco is an online simulated ecosystem game developed by Strange Loop Games, funded by the United States Department of Education (IES, 2015; Strange Loop Games, 2018b) and an online crowdfunding campaign (Kickstarter, 2015). The game’s main objective is to stop a giant meteor from crashing into the surface of the earth, which is set to strike after a fixed time period of 30 real-life days (Meteor, 2018). While developing the requirements for stopping the meteor, players also cause pollution which needs to be minimized so that the ecosystem can continue to thrive – a measure of which can be found in an in-game statistical overview available to the players. In other words, the players need to destroy the meteor as well as maintain balance in the virtual ecosystem that the game provides them with.


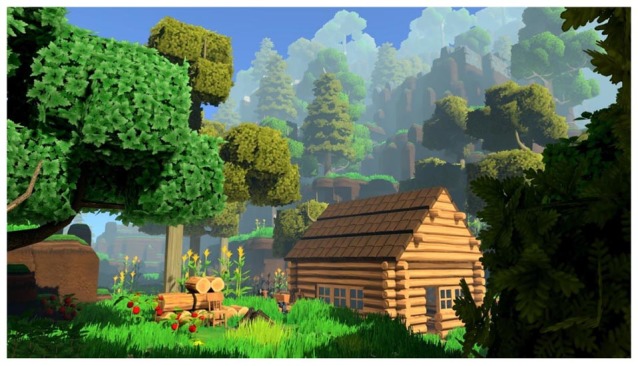


**Example of a player-generated house from Eco, as well as the game’s user interface (UI). Note the stacked wooden logs in the left of the picture, used by the players to make a variety of in-game structures. Image used with permission.**

### The Effectiveness of Game-Based Learning

Virtual environments, such as educational games, constitute promising new research tools in various kinds of environmental behavior research (de Kort et al., 2003), and have demonstrably been shown to alter behavior in real-life settings. Educational games are also receiving increased attention within the field of sustainability education and conservation (Sandbrook et al., 2015). Here, researchers focus on topics ranging from the effects of environmental change on marine ecosystems (Ghilardi-Lopes et al., 2013) to knowledge of energy use (Yang et al., 2016) and sustainable land management (Schulze et al., 2015). Sustainability games are used in order to make intangible environmental issues more salient, although the learning outcomes from playing them vary (Boomsma et al., 2018). On the positive side, one study revealed a significant correlation between experiencing a high degree of *game enjoyment* while playing a game about local biodiversity (*BioDiv2Go*) and a subsequent increase in attitude toward nature (Schaal et al., 2018). Enjoying environmental gameplay is theorized to have a significant effect on the subsequent learning outcomes from playing (Fjællingsdal and Klöckner, 2017), thus lending support to the study’s findings. Another study revealed that individuals who played Red Redemption’s *Fate of the World*, a simulation revolving around a 200-year period of societal and environmental impacts (Klöckner, 2015, p. 198), showed a higher degree of environmental *systems thinking* than a control group (Waddington and Fennewald, 2018). Systems thinking – the ability to understand the complexity of all the individual parts of an interconnected system (Aronson, 1996) – is crucial in the understanding of ecosystems.

### Environmental Consciousness, Personal Responsibility and Environmental Action

*Environmental consciousness* is the measure of a person’s overall environmental concern, the degree to which they believe that threats toward the environment pose an urgent and immediate problem to their everyday lives (Schlegelmilch et al., 1996). It is a multifaceted psychological construct consisting of cognitive aspects such as knowledge, values, concerns and problem awareness on one end, and vicarious and direct experiences with environmental issues on the other (Kollmuss and Agyeman, 2002; Sánchez and Lafuente, 2010; Sarrica et al., 2016). Environmental consciousness also incorporates an individual’s overall level of *environmental awareness* – a general state of alert and understanding of one’s impact on the environment (Grob, 1995; Sarrica et al., 2016) – as well as *environmental concern* – negative affect and beliefs about environmental problems (Schultz et al., 2004). An individual’s degree of environmental consciousness is dependent on the prevalence and interconnectedness of each of these facets. In practice, this means that a high degree of environmental knowledge, for example, is seldom enough to initiate pro-environmental action on its own (Hines et al., 1987; Kollmuss and Agyeman, 2002; Frick et al., 2004; Abrahamse et al., 2007). However, when paired with other environmental consciousness facets such as behavioral intent and affective components, knowledge can be highly efficient as a driver toward pro-environmental behavior (Secord and Backman, 1964; Stoknes, 2017, p. 90). Previous research has demonstrated the effectiveness of a high degree of interconnected environmental consciousness factors. One study, for instance, showed that feelings of personal responsibility, combined with environmental knowledge and environmental values, accounted for 76–94% of ecological behavior (Kaiser et al., 1999).

While the promotion of environmental consciousness is highly important in order to circumvent the growing number of environmental issues threatening the globe, the level of concern is declining in certain countries – despite the scientific consensus that climate change and other sustainability issues are on the rise (Cook et al., 2013). In Norway, the percentage of the population believing climate change to be one of the three biggest contemporary societal issues went down from 34 to 25% between 2015 and 2016 (TNS Gallup, 2016). Some numbers, however, specify that approximately 97% of the Norwegian population is knowledgeable or aware of climate change (Pelham, 2009) and that the country has a high degree of political emphasis on environmental education (NOU, 2006, 2010, 2014). This illustrates that while the degree of knowledge and problem awareness of climate change might be high, other environmental consciousness factors such as concern or direct experience might be low – thus leading to an overall low level of environmental consciousness (Sarrica et al., 2016). While being aware of an environmental issue is seldom enough to initiate pro-environmental action, understanding the link between one’s own actions and subsequent environmental decline could lead to pro-environmental behavior (Hines et al., 1987). According to the *stage model of self-regulated behavioral change*, an important precursor to pro-environmental action is a feeling of *personal responsibility* for the environment – which also entails being conscious of how one’s actions negatively impact nature (Bamberg, 2013). Such personal ecological norms are shown to predict pro-environmental behavior such as sustainable travel mode choices (Hunecke et al., 2001) and the preservation of marine environments (Cottrell and Meisel, 2003).

### Immersion and Flow – Directed Attention and Intrinsic Motivation

Educational games must be perceived as enjoyable or *immersive* by the player in order to be voluntarily used (Sweetser and Wyeth, 2005; Ferguson and Olson, 2013; Fjællingsdal and Klöckner, 2017; Hamari and Keronen, 2017) or, despite undermining intrinsic motivation to play and learn, offer some form of externalized reward such as money or extra course credit (Deci, 1996, p. 25). Immersion, otherwise known as *presence*, is the feeling of being spatially present in a media experience (Klimmt et al., 2009). When immersed, the player is absorbed and engrossed in the progression of a game, and their attention is often directed entirely toward the game itself (Brown and Cairns, 2004). A high degree of immersion in virtual content can increase scores on connectedness to nature, which is shown to lower the prevalence of self-focused values and value-laden behaviors (Weinstein et al., 2009). It is also an indicator that the game is intrinsically motivating to play (Przybylski et al., 2010).

Immersion is considered a precursor to the *flow* concept, where a task is perceived as an intrinsically motivating experience (Csikszentmihalyi, 1990, p. 1). If a game is not immersive, it likely won’t be played voluntarily (Brown and Cairns, 2004; Sweetser and Wyeth, 2005). Immersion and flow are important for an individual’s desire to interact with a game, and a high degree of immersion during gameplay has been shown to increase a player’s *suspension of disbelief* (Cheng and Cairns, 2005) where a person overlooks realistic flaws in media in favor of an enjoyable experience (Wirth et al., 2007; Böcking, 2008).

In contemporary literature descriptions, immersion builds as the media user forms a mental representation of the space or world that the media experience seeks to provide, whereupon it becomes subjected to a variety of *individual factors* that either strengthen or break it (Brown and Cairns, 2004) – ranging from the user’s degree of involvement in the media experience to their perception of how realistic it is (Wirth et al., 2007). Some researchers also suggest that immersion is gender-based, where *males* tend to be more attracted toward fantasy elements as well as the ability to compete with their social peers (Chou and Tsai, 2004). *Female players* on average tend to play less than men (Hartmann and Klimmt, 2006) and generally refrain from playing competitively (Wood et al., 2004). Female players instead prefer games allowing for meaningful social interaction (Hartmann and Klimmt, 2006) and emotional experiences (Schell, 2008). Furthermore, clear progression goals and feedback from the game as well as continuously increasing difficulty are important for the overall gameplay experience (Schell, 2008).

### Feedback and Eco-Visualization

In Eco, the consequences of the player’s actions become *eco-visualized* (Löfström and Svanæs, 2017) – trees and water supplies get visibly polluted when waste materials are incorrectly stored (Tailings, 2019), and toxic water turns an abnormal color (Strange Loop Games, 2015). This visualization constitutes a core factor in *feedback*, a central element in both *game design* (Schell, 2008) and *environmental communication* (Abrahamse et al., 2007). In game design, feedback provides the players with information on how they are progressing within the game (Sweetser and Wyeth, 2005; Fu et al., 2009), usually by giving them information on where they are going next or what their current goals are (Schell, 2008). In addition to steering the player’s actions, in-game feedback is also significantly related to the enjoyment of the game (Sweetser and Wyeth, 2005). In environmental communication, similar feedback interventions tend to provide information about measurable changes in someone’s ecological footprint, such as decreases in energy use (Abrahamse et al., 2007).


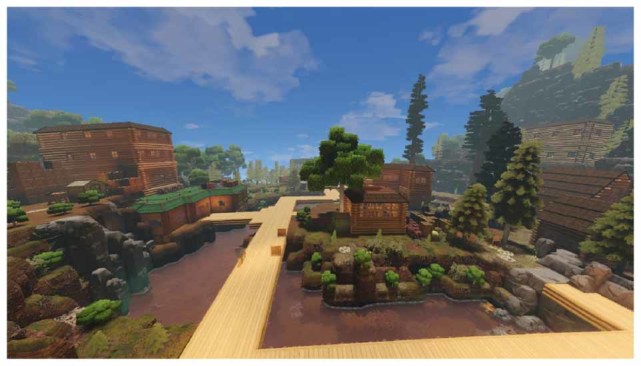


**Example of a player-generated base society in Eco. Note the pinkish water – one of the indications that it is polluted. Image used with permission.**

### Goal Framing and Tragedy of the Commons in Eco

According to Goal Framing Theory (GFT), maximizing one’s pleasure both in the present (*hedonic goals*), securing a comfortable and secure future (*gain goals*) and acting appropriately in a group (*normative goals*) are central motivators for behavior (Lindenberg and Steg, 2013). A player in Eco is free to gather resources for themselves, thus fulfilling their hedonic and gain goal needs, but they are also required to share resources with others in their group as well as replant and replenish the resources they consume. Should they fail to do this, other players will not gain access to the resources they need and will not be able to progress in the game.

Furthermore, actions in Eco cost *skill points* that are acquired through a varied diet as well as having a fully furnished home (Skill Points, 2019). A server where resource hoarding is occurring will lead to other players being unable to perform important actions. This simulates the *Tragedy of the Commons*, an occurrence in shared-resource systems where several actors seek to maximize their own gains, usually resulting in a lack of resources for the group as a whole (Hardin, 1968). If a large group of people gather as many resources as they can without replanting or renewing them, the environment will inevitably collapse and become barren. An illustrative experiment on a finite resource dilemma using a fishing simulation revealed that players generally exhibited restraint in their consumption when the fish population was perceived as critically low and that individuals with more pro-environmental values fished less than the other players (Sussman et al., 2016).

### Ecosystem Complexity and Systems Thinking in Games

As previously described, Eco simulates a digital ecosystem in which the players must cooperate in order to maintain balance. An *ecosystem* is a complex, adaptive and often non-linear or chaotic system (Fiksel, 2006) consisting of components that are vital for life on Earth (Tansley, 1935). Ecosystems and the biota contained within regulate and enable processes necessary for biological life, such as the sequestering of harmful chemicals and mediating climactic and atmospheric processes on a global level (Levin, 1998). A healthy ecosystem has the ability to remain structured, organized and functioning even when subjected to external stress, which involves numerous complex interactions between its individual components (Costanza and Mageau, 1999). Ecosystems can be resilient, but scientific evidence overall suggests that human activity is severely impacting biological systems on a global level (Rosenzweig et al., 2008). Damages to the ecosystem have been shown to lead to a wide variety of *biodiversity loss*, a reduction in the number of species necessary for maintaining the processes enabling biological life (Loreau et al., 2001; Worm et al., 2006). Ecosystem protection is therefore of great importance, but it is also a highly complex topic where each system component is vital to ecosystemic functioning.

While the interconnectivity of the processes in an ecosystem can be difficult to understand, there are some pedagogical approaches to it that have shown promising results. One such approach is known as *systems thinking* – the ability to see a complex entity as a whole (Checkland, 1999, p. 50). Systems thinking might increase knowledge about how ecosystems function (Frick et al., 2004). It has been shown that simulations and games are highly suitable for teaching about the complexity of systems, and that some games have been developed specifically to address environmental topics such as climate change (Waddington and Fennewald, 2018).

### Cooperation in Sustainability and Eco’s Profession System

One of the central barriers against pro-environmental behavior is the feeling that individual efforts alone do not lead to change (Stoll-Kleemann et al., 2001; Aitken et al., 2011; Axon, 2017). A common reasoning for this is that environmental issues are global, and that there is therefore little point in individual action (Gifford, 2011). Individuals who *do* engage in pro-environmental behavior overall tend to practice values *beyond* the interests of the self (Steg and Vlek, 2009), such as participating in groups to perform civic engagement or joining environmental organizations (Hamilton et al., 2018). Group membership is also important for developing an individual’s *values*, which in turn shape much of our intrinsic motivation to perform some sort of pro-environmental behavior (Kollmuss and Agyeman, 2002).

Eco has a strong focus on cooperation (Getting Started, 2019), and players need to form groups in order to maintain balance in their simulated ecosystem. This mechanic sets it apart from more traditional zero-sum games, where competition, sabotage and fighting results in only one clear winner (Fennewald and Kievit-Kylar, 2013). Each player on a server picks a profession and develops it by acquiring role-based skills (Professions, 2019). Each profession is important for the maintenance of the ecosystem the players live in, and cooperation between the professions is required in order for the game to progress. A *hunter*, for example, needs to fetch meat in order for the *chef* to cook food for the group. The chef receives crops from the *farmer*, which improves food quality. Food helps players perform activities, like the *smith* developing metal ingots for the *engineer* to utilize in various constructions.

## Materials and Methods

### Recruitment and Participants

The respondents were recruited from two Norwegian high schools and three Norwegian university classes as well as four Facebook groups affiliated with the subjects of environment and games. Students in the high school- and university classes received information about the project through lectures, while the Facebook groups received a digital document containing the details of the study. In this document, the respondents were introduced to Eco and the purpose of the research project. They were also informed that they would receive an invitation for a voluntary post-gameplay interview about their experiences once the gameplay sessions were concluded. Once the initial recruitment procedure was finished, some respondents recruited others through *snowball sampling* for six additional participants. A total of 59 individuals agreed to receive a copy of the game for testing. 46 of them (77.9%) were male. The age range of the respondents varied from 18 to 31 years, and 36 (60%) of them were between 16 and 20 years old. The majority of our respondents had previous experience with video games, with a large part of the sample noting that they had played video games actively since childhood. 57 (96.6%) of the respondents reported previous experience with videogames, with 24 (40.6%) of them listing themselves as having played videogames for more than 15 years. Only 3 (5%) individuals had never played videogames before they played Eco. The respondents also appeared to be active gamers, with a majority of 42 (71.1%) of them playing videogames for more than three times a week. 37 (62.7%) respondents stated that they played videogames for more than 3 h per day. They also noted that despite being conscious of environmental issues, they did not always adjust their behavior to circumvent them and would rather perform commonly practiced pro-environmental activities (Table 1) that require relatively little effort, such as recycling (Hamilton et al., 2018). Of the 59 individuals who received a copy of the game, 7 (*n* = 7) agreed to participate in the qualitative post-gameplay interviews with the lead researcher.

**TABLE 1:** Active pro-environmental actions performed by the respondents.

**Respondent ID**	**Recycling or clearing trash**	**Reducing food waste**	**Biking or public transportation use**	**Using cloth bags instead of plastic bags**	**Taking shorter showers**
R1, age 28	X				
R2, age 21	X				
R3, age 29	X		X		
R4, age 24	X	X		X	
R5, age 25	X		X		
R6, age 18			X		
R7, age 19	X				X

### Instruments and Experimental Procedure

Before the study was initialized, all respondents were given access to Eco through a unique 5-digit user-ID and 4-digit password. Eco was in *beta stage* at the time of the study, meaning that the game was nearly complete but not yet ready for an official release (Beta, 2018). 100 unique user accounts were made available to the lead researcher through the purchase of the *Eco Classroom Pack* (Strange Loop Games, 2018c) before recruitment started. These user accounts were distributed among the respondents with instructions about how to play the game. The respondents were encouraged to recruit other players if they wished. The lead researcher’s e-mail was also provided, in case the respondents encountered any technological errors while they played.

Once the respondents had finished their gameplay after 2–4 weeks, the lead researcher interviewed them about their in-game experiences. Qualitative interviews were chosen as an information gathering strategy due to the potential quality of the insight they might provide (Wainwright, 1997), even for smaller samples of respondents (Crouch and McKenzie, 2006; Fugard and Potts, 2015). seven respondents agreed to participate for an interview, all of which were male. six respondents were interviewed online through *Skype* or *Appear.in*, whereas one respondent filled out the interview guide manually in a Word document. Each interview lasted between 30 min and 1 h. The interviews were recorded with the *SnagIt* screen capture software, and the respondents all gave their consent to be recorded. The interview guide was made by the lead researcher and consisted of 10 open-ended questions primarily centered on Eco’s educational content (Table 2). The participants were instructed to answer each question as honestly as possible and were ensured that the information they provided would be of great assistance to the researchers – a type of questioning considered ideal for the *quality testing of games* (Schell, 2008). Due to the population sample’s national background, the questions were asked in Norwegian.

**TABLE 2:** Interview questions.

Do you consider yourself an environmentally conscious person?
What are your thoughts on using games like Eco in an educational setting?
Do you feel that you have learned something about the environment from playing Eco?
Is there anything about Eco you would describe as particularly good?
Is there anything about Eco you would describe as particularly bad?
Could you describe how you felt while playing Eco?
Do you feel that Eco has changed your view of the environment?
Do you feel that Eco has taught you something about how to circumvent environmental issues?
What are your thoughts about the level of difficulty in Eco?
Do you have any other thoughts or comments about the Eco project?

### Analysis Procedure

**FIGURE 1:**
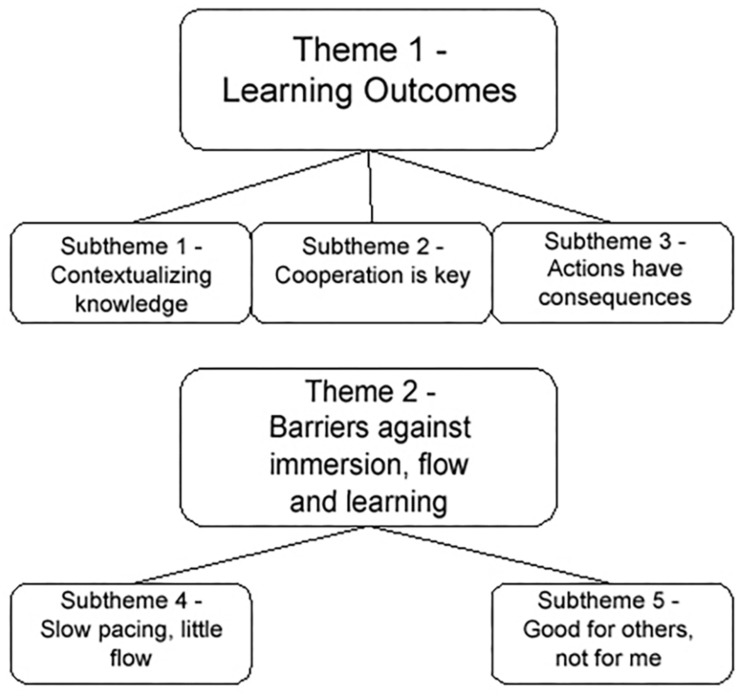
Complete thematic map.

Once the recorded interviews had been transcribed, a thematic analysis based on Braun and Clarke’s (2006) framework was conducted by the lead researcher. This was done in six steps: (1) data familiarization, (2) coding, (3) initial thematic categorization, (4) thematic review, (5) thematic naming and definition, and (6) article writing. In the *data familiarization stage*, the lead researcher got acquainted with the existing data sets. Answers from the respondents containing vital information to the research project were then extracted and highlighted using appropriate tools in Adobe Reader and listed as *codes* for later thematic categorization. Recurring answers that signified agreement or opposition between the respondents surrounding one of the interview’s main topics were then categorized in a document, serving as *initial thematic categories*. These were then subjected to a *review* from the lead researcher, who established a thematic map of the final thematic categories (Figure 1). These were then subsequently *named* and illustrated with direct quotes from the respondents.

### Ethics Statement

All of the respondents in the study were provided introductory material about the game and the purpose of the research being conducted. The project was reported to NSD – Norwegian Centre for Research Data, and the respondents were provided with a draft of the article for informant validation. Each informant was given 7 days to provide feedback on any misquotations that the lead researcher may have made.

## Results

### Theme 1 – Learning Outcomes

The core idea behind a serious game is its ability to teach something to its players (Wouters et al., 2013; Fjællingsdal and Klöckner, 2017) through providing the players with new knowledge, raising awareness for something or presenting research findings in a novel way (Eisenack and Reckien, 2013). The main theme detailing our respondents’ learning outcomes contains three subthemes: (1) *Contextualizing knowledge*, (2) *Cooperation is key*, and *(3) Actions have consequences*.

#### Subtheme 1 – Contextualizing Knowledge

“I would say that Eco reminded me that my actions have consequences, and that humans need to try fixing pollution together. Technology can help us save the planet, but eventually we need to do something.” (Respondent 2, age 21)

“I didn’t get far enough to learn anything particularly new, but I quickly noticed that not everyone could build houses as big as they wanted and how dividing resources is challenging when everyone’s got their own projects of equal importance going on.” (Respondent 4, age 24)

“I have gotten to feel what happens when you overload the environment – you feel it a bit more by testing it out rather than just hearing about it. It’s very abstract, but when you get it simulated through Eco then you see it a bit more clearly.” (Respondent 5, age 25)

“I was gathering food and thought ‘what is easily accessible that doesn’t cost me a lot of time so I can work on other stuff? Fish and blue mussels!’ So I spent about 2 h gathering those. But then I saw that seaweed in the ocean just dropped down 2-3000 due to how I had continuously been gathering for 2 h. So if you can imagine 100 people doing the same as I did there, there wouldn’t be any life left. It’d just go straight down.” (Respondent 7, age 19)

Environmental knowledge is a central determinant for pro-environmental behavior (Hines et al., 1987; Kaiser et al., 1999; Abrahamse et al., 2007). Norway has a strong political emphasis on environmental education (NOU, 2006, 2010, 2014), which likely contributes toward the population’s high level of environmental awareness (Pelham, 2009). A result of this is that playing Eco did not teach the respondents anything new about ecosystems, but instead served to *reinforce their existing knowledge* or as a reminder about contemporary issues related to bio-conservation. This reinforced knowledge stems from the way the game presents information and makes this information salient by establishing a concrete link between the players’ actions and resulting environmental change – which is of great importance in regard to generating a variety of pro-environmental behaviors (Kaiser et al., 1999; Hunecke et al., 2001; Cottrell and Meisel, 2003; Bamberg, 2013). Respondent 2 mentions that Eco has reinforced his belief that technological development, or *technosalvation* (Gifford, 2011), is not enough in order to circumvent climate change and that human action is required. Respondents 4 and 7 discussed their experiences with how Eco presents the *Tragedy of the Commons*, or the notion that everyone in a shared-resource ecosystem will suffer if one or several parties overuse resources (Hardin, 1968). Respondent 4 mentioned how his team had to set aside their individual-centric hoarding behavior in favor of sharing resources equally among the server population, suggesting that their goal framing shifted from self-centered and hedonic to group-friendly and future-oriented, as described by GFT (Lindenberg and Steg, 2013). Respondent 5 states that while he felt he didn’t learn anything particularly new from playing Eco, he did refresh his understanding of how ecosystems work – suggesting reinforcement in his systems knowledge (Frick et al., 2004). Another finding of interest was the story told by Respondent 7, who single-handedly managed to overfish his server’s population of fish and shellfish, illustrating how Eco is capable of making environmental issues such as *overfishing* (Sussman et al., 2016), *abnormal alterations in oceanic life* (Lejeusne et al., 2009) and *biodiversity loss* (Loreau et al., 2001; Worm et al., 2006) salient. Intriguingly, Respondent 7 is the only individual in our study specifically mentioning the in-game *statistical overview of existing species* that Eco provides, indicating the importance of highlighting and informing the players about this particular tool for future playing sessions. He was also the most prominent in describing how Eco made him think about the interconnectivity of a complex ecosystem (Tansley, 1935; Costanza and Mageau, 1999; Fiksel, 2006; Rosenzweig et al., 2008), thus suggesting an increase in his level of systems thinking (Checkland, 1999; Frick et al., 2004).

#### Subtheme 2 – Cooperation Is Key

“I started by playing by myself, but it quickly became too large and too complex. I can’t remember how exactly it happened, but suddenly we were five! We set up our own server where we are still playing, where we have one carpenter and one blacksmith and a farmer and an engineer and a hunter with some overlap. You notice that it becomes a completely different game. When you cooperate and plan with others and you ask others for help and you get synergy effects between jobs… it is really fun and engaging.” (Respondent 3, age 29)

“A challenge for the group I played with was progressing in skillpoints and such (…). Other than that it was a very fun social activity and it managed to make us quarrel about how much wood we were allowed to use in order to build our houses, since we quickly realized that there wasn’t enough materials in our immediate vicinity for us to gather efficiently until we got carts to carry them in.” *(Respondent 4, age 24)*

“The game looks like it is intended for other people to become part of your world, especially considering the politics system of the game. But since I played alone, I had no need for politics or cooperation with others. So… there wasn’t really a happy feeling. Because the game doesn’t have one of those ‘if you do something good you get something good’ – it doesn’t have that reward system. (…) The game is based on how you can cooperate with others. But it also has a very negative angle on how one person wants everything. So I would say that, yeah, it has changed my view on that a bit.” (Respondent 6, age 18)

Ecosystems management requires interdisciplinary insight and collaborative effort in order to be successful (Salafsky et al., 2002). Research also shows that one of the biggest barriers against pro-environmental behavior is the feeling that individual efforts are insufficient to combat climate change (Stoll-Kleemann et al., 2001; Aitken et al., 2011; Gifford, 2011; Axon, 2017). Collaborative action was conducted by the majority of the study sample during gameplay of Eco; 6 out of 7 respondents described Eco as a game that you had to play with others in order for it to be enjoyable. Respondent 3 tried playing Eco alone but got overwhelmed by its complexity, mirroring the notion in scientific literature that ecosystems are highly complex constructs (Tansley, 1935; Costanza and Mageau, 1999) and that interdisciplinary cooperation is required in order to manage them (Salafsky et al., 2002). He notes that once he understood how the professions in Eco are interdependent, he experienced a boost in his gameplay enjoyment. Respondent 4 and his team realized that their server featured limited resources and debated how to share them. It is possible that Respondent 4’s group developed a shared value where limited resources were important for the group’s survival, showcasing how pro-environmental group-based values [as described by Kollmuss and Agyeman (2002)] can occur in games. It also suggests that that perceived scarcity of resources in games leads to more cooperative behavior among members of a group (Sussman et al., 2016). Respondent 6 played the game alone and described the experience as rather negative, citing what he perceived as a lack of feedback from the game. His actions did not lead to tangible rewards such as becoming stronger or understanding the game’s next objective. This type of feedback is almost universally considered to be an important game enjoyment factor (Sweetser and Wyeth, 2005; Schell, 2008; Fu et al., 2009).

#### Subtheme 3 – Actions Have Consequences

“I started out with a pretty solid understanding (of the environment), but it was interesting to see that when a large group of people arrived, the environment suffered. So it just reinforced what we already have a theory about, if you have ever opened a book on natural sciences. The more people there are, scraping the area for resources, the less careful they are about making them grow again.” (Respondent 1, age 28)

“The fact that you have a very limited amount of space for carrying stuff, when you are chopping trees for example, you can’t really just bring the entire tree with you back to your base – you have to go back and forth, back and forth and fetch the resources. It makes it feel like you are emptying it more. You really feel how much you are actually collecting, versus Minecraft where you just chop and chop and chop and then suddenly you have thousands of resources. You feel how much you collect, due to the amount of work that takes.” (Respondent 5, age 25)

“(…) Instead of just gathering resources haphazardly, your actions had a visible effect on the environment. I think that, Minecraft could have a thing where if you cut down a bunch of trees then nature could get worse – I think it makes you become a bit more interested when your environment changes because of something that YOU do.” (Respondent 6, age 18)

Eco depicts the consequences of the players’ actions on their surrounding environment, a strategy commonly used in eco-visualization (Löfström and Svanæs, 2017). It shows environmental decline through plants turning brown, crops and animals disappearing, the ground becoming barren once a fallen tree lands on it and water turning a pinkish hue (Strange Loop Games, 2015). Pollution in Eco is a sign that the player is doing something wrong and that they need to prevent similar issues in the future, such as by burying mining Tailings (2019). Several of the respondents became aware of these environmental changes while playing, and the visual depiction made them feel as if their pre-existing knowledge of environmental issues had been reinforced. Respondent 1 describes himself as familiar with environmental topics, and that a simulated version of environmental issues and how they develop as a consequence of resource overuse was an interesting experience. Respondents 5 and 6 draw comparisons between Eco and Mojang’s *Minecraft* from 2009, a game centered around building and developing structures and items from various materials. In Minecraft, players can carry near-unlimited amounts of materials. In Eco, the amount of resources a player can carry is limited in order to reflect a more realistic resource gathering situation. Respondent 5 mentioned that this made him aware of how much he was affecting the environment by being given a visual and affective depiction of his own actions. Respondent 6, while feeling restricted by the game’s mechanics, also mentions that it was interesting to get these visual depictions.

### Theme 2 – Barriers Against Immersion, Flow and Learning

Despite the potential educational benefits of playing Eco, our analysis also revealed that the game contained elements that had an overall negative impact on the players’ degree of immersion, flow and learning outcomes – described here as barriers. The construction of this theme revealed two subthemes: (1) Slow pacing, little flow and (2) Good for others, not for me.

#### Subtheme 4 – Slow Pacing, Little Flow

“We always ended up in the situation where one person had to sit and wait for one of the other players for them to get skill points to progress and make something needed to progress. (…) … you freeze COMPLETELY if you don’t cooperate. The issue was that since we were only 5 people, this was difficult to implement. We talked about how we should have been 10 – 20 people, then we would’ve gotten more out of the game – we were simply too few. (…) We tried to tweak the settings a bit in order to adjust how many skills we got, but we never found the sweet spot – it either went too slowly, or too quickly.” (Respondent 2, age 21)

“The way the skill system works is that you are supposed to have a big server going and the 30 days before the comet hits are actual real-life days. (…) The skill system is what allows you to choose what to learn and do in the game, and it is dependent on time and what food you eat and what house you have. It is interesting, but in practice it works poorly when there are few players. (…) 30 real-life days is a long time to experience the comet if you don’t have a big server to play on. That said, they do have pretty good systems for adapting these factors – you can control when the meteor is coming, you can turn it on and off, so… their server tools are nice like that, like, they make the players do it.” (Respondent 5, age 25)

”I think it would get a bit difficult to just sit down and play this, and use a lot of time – because that is what Eco is doing now. With skillpoints, in order to learn stuff, you must be a member of a specific server so and so many days. And the timeframe for starting the server anew is 30 days, so you start from scratch on day 1 and by day 30 you must reach the endgame.” (Respondent 7, age 19)

In educational games, the importance of immersion and flow is frequently highlighted (Brown and Cairns, 2004; Sweetser and Wyeth, 2005; Jennett et al., 2008; Fjællingsdal and Klöckner, 2017), and should the game somehow fail to induce these psychological states in its players it is likely to negatively impact the players’ learning outcomes. Immersion and flow are both easily broken, such as through faulty level design or a lack of concentration on the game (Brown and Cairns, 2004; Schell, 2008). A lack of flow leads to frustration and boredom – psychological states that players normally wish to avoid by playing games in the first place (Ferguson and Olson, 2013). As previously mentioned, Eco’s gameplay takes place over a period of 30 real-life days (Meteor, 2018). In contemporary research literature, this is known as slow serious games – educational games designed to deliberately allow the player a very limited timeframe to progress. The intention behind this is to provide the player with ample opportunity to reflect, contemplate and learn from their in-game actions (Marsh, 2015). For several of our respondents, this design was perceived as too lengthy for an enjoyable gameplay experience. While there is little consensus in contemporary literature in regard to how long a game should be, our respondents felt “forced” to play it for 30 days consecutively due to how the gameplay session never ceases to progress – even when the players are offline. They also normally composed smaller teams of four to five individuals, whereas established Eco servers can have significantly larger populations. Respondent 2 points to how some of the players on his server had to wait for others to gain skill points in order to make progress, which was not feasible due to how small their group was and how interdependent the individual members were. Respondents 5 and 7 also mirror this notion, with Respondent 5 mentioning that the game can be adjusted and configured to fit the individual player. Respondent 2 made an attempt at this during his gameplay sessions but was unable to properly configure the game to his group’s needs. It would appear that the respondents felt an overall lack of control over the game’s rules and boundaries, which negatively impacted their sense of flow.

#### Subtheme 5 – Good for Others, Not for Me

“For me it didn’t do much – but that likely has to do with how I paid attention to science class. But I won’t exclude the possibility that it might do something for very many others, since this tends to be a rather boring topic for many people. Not because they are not interested, but because cause and effect is very abstract for people. If you remove everything the rabbits eat, then the rabbit has nothing to eat and the wild rabbit population in Norway dies out. For them, this seems to be such a distant reality that it appears irrelevant.” (Respondent 1, age 28)

“For some it might be effective, but… for me, who holds an above average interest in videogames, I can’t really avoid “looking under the hood” [of the game] and recognize “oh, this is how that works, that was fun, that was a cool way to implement pollution in the game”.” (Respondent 3, age 29)

“I have read a lot about the environment, so I don’t feel like I have learned anything new. I think very young people can play this game, but I assume that many adults already know that this is happening in nature.” (Respondent 6, age 18)

Norway has a large focus on environmental literacy education (NOU, 2006, 2010, 2014). Perhaps as a result of this, several of our respondents experienced that playing Eco did not increase their environmental knowledge to any significant degree. They did, however, express that using Eco to teach *new learners* about environmental topics could be a possible future implementation strategy. Respondent 1 states that he has experience with environmental education from before but highlights the importance of fun and playful approaches to learning about unfamiliar or tedious subjects. This mirrors an overall tendency in the use of environmental games to promote learning – they can be fun and engaging despite their overarching topic (Klöckner, 2015, p. 198). For Respondent 3 it appears that his interest in the game’s mechanics and coding was significantly stronger than the emphasis on teaching about the environment, suggesting that a person’s mindset and priorities during gameplay will impact the educational benefits of playing Eco. Respondent 6 mentions that despite what he perceives as a narrow target audience, Eco might be capable of teaching younger individuals about the environment.

## Discussion and Conclusion

The purpose of this thematic analysis has been to examine how playing Eco might promote environmental consciousness surrounding ecosystems. Our results suggest that Eco has the potential to reinforce and increase some facets of environmental consciousness by visualizing the impact of human activity on ecosystems in a novel way, although the majority of our respondents did not engage with the game. Additionally, a significant amount of respondents declined to participate in post-gameplay interviews. In the first part of this discussion section we will analyze the more promising aspects of Eco’s role in sustainability education. In the second part we will consider and analyze the low response rate after the gameplay sessions, as well as the apparent lack of motivation to engage with the game itself.

Overall, our findings add to the growing body of research suggesting that educational games constitute a promising and novel way of learning (Wouters et al., 2013), also mirroring the research done by previous sustainability researchers utilizing games (e.g., Schaal et al., 2018; Waddington and Fennewald, 2018). One of the central findings from our study is that Eco has been shown to reinforce and contextualize our respondents’ overall level of environmental literacy and systems thinking. These are highly important skill sets (Fiksel, 2006) that could result in a greater understanding of ecosystem complexity, i.e., how different biomes interrelate and interconnect with one another, or how certain species are interdependent in a cyclic system. Our results show that Eco appears to be capable of visualizing the complexity of an ecosystem in a way that allows its players to comprehend and conceptualize the interconnectivity and balance that exist in nature, as well as the actions that upset or maintain this balance – i.e., that actions have consequences. This level of understanding occurred, at least for some of the study’s participants, over a wide range of contemporary ecosystem vulnerabilities – such as *overfishing* (Sussman et al., 2016), *abnormal alterations in oceanic life* (Lejeusne et al., 2009) and *biodiversity loss* (Loreau et al., 2001; Worm et al., 2006).

Perhaps due to the game’s ability to visualize otherwise intangible subjects for its players, there is evidence to suggest that playing the game has an impact on environmental consciousness. Going by the definition of environmental consciousness as a multifaceted psychological construct (Sarrica et al., 2016), there is evidence that some of our respondents show a slightly elevated level of environmental awareness. Environmental awareness, a general state of alert and understanding of one’s impact on the environment (Grob, 1995; Sarrica et al., 2016), could clearly be identified in some of the vivid experiences illustrated in the subsections of Theme 1 – especially in regard to the game’s visualization of personal impact on the game world. Added to the fact that there is a significant degree of political emphasis on environmental education where the study took place (NOU, 2006, 2010, 2014), there is a significant likelihood that other cultures might also benefit from playing Eco.

Eco also showcased the effects of game-based eco-visualization and cooperation. Games are generally voluntary and pleasurable activities (Sweetser and Wyeth, 2005; Ferguson and Olson, 2013; Fjællingsdal and Klöckner, 2017; Hamari and Keronen, 2017) but can also be highly educational. Eco visualizes the effects of anthropogenic climate change in the same vein as past eco-visualization research (Löfström and Svanæs, 2017). Another interesting aspect of Eco is its strong focus on cooperation in counteracting sustainability issues (Getting Started, 2019), which appears to have been fully understood by the majority of our respondents – even those who chose to play alone. Judging from our results, Eco represents an innovative and promising classroom tool for showcasing and contextualizing how group-based activity and behavior can counteract threats to our environment. Added to our findings that Eco is capable of increasing systems thinking and reinforcing existing knowledge about the environment, it is a valuable tool for future environmental education.

The thematic categories in our study did, however, end up being very broad. This is to be expected due to the nature of Eco’s mechanics. Eco emulates an entire ecosystem, where each individual theme and facet is interconnected. Focusing only on individual facets would result in the players “missing the bigger picture” as described by Kollmuss and Agyeman (2002). As a result, the players are forced to consider each individual aspect of the ecosystem in order to play the game effectively. The players cannot, for example, go around wiping out various animal species, as this will lead to potential food shortage. They also cannot put mining tailings everywhere, as these will eventually seep into their water supply, poisoning it. They also need to be mindful of their resource use, replanting trees, carbon emissions and the rate of technology development – all on top of considering the different needs in their group. Altogether, this illustrates how much the players need to consider simultaneously. This variety of topics enables the players to engage in *systems thinking*, or the ability to see a complex entity (i.e., an ecosystem) as a whole (Checkland, 1999, p. 50) rather than just the “sum of its parts.” A narrow focus on only specific topics in Eco might result in losing the vision that the game wishes to simulate – the complex interconnectivity of an ecosystem. Also, due to the use of a semi-structured interview guide with open-ended questions, the freedom experienced by the respondents left them with a lot of room to answer, and their replies were almost certainly guided by their own unique experiences. As a result, some players will experience the water pollution of mining tailings, while others will experience the issue of a lack of food to generate skill points. This leads to a variety of different experiences that, consequently, also leads to the generation of wide categories of information.

Despite encountering game mechanics issues common for games in the beta stage, most of our respondents described Eco as an interesting experience. It is worth noting, however, that future researchers wanting to implement Eco into their research need to be aware of these implementation issues. *Firstly*, Eco takes place over a period of 30 days – it therefore needs to be well-planned and well-informed so that the players do not disconnect from the experience during the experiment. *Secondly*, it is crucial to give the players a general introduction to the controls and overall purpose of the game to avoid any confusion and lack of flow during gameplay. *Thirdly*, organizing a debriefing session once the game is over, where the players can clear up any misconceptions they have made during their gameplay as well as to have a scientific discussion about the game’s many overarching topics, is warranted. Keeping these considerations in mind could improve the gameplay experience for the respondents, and clear any misconceptions they might have.

While our study demonstrates that Eco does hold some promise in regards to its utilization as an educational tool for environmental consciousness, the recruitment procedure yielded a surprisingly low number of respondents from the high schools. As a result, only respondents from the Facebook groups and university classes participated in the post-gameplay interviews. As a compound issue, none of the interviewed respondents were female. A possible explanation for the lack of respondents from the high schools is that the planned gameplay sessions took place right before the Norwegian high school winter exams. Due to Eco’s 30-day forced play cycle (Meteor, 2018) and overall complexity, it is fair to assume that the students simply did not prioritize playing the game over studying for their finals. Curriculum time pressure has been identified as a central barrier in the implementation of educational games in the past as well (e.g., Lim et al., 2006). Added to the fact that playing Eco did not yield any tangible externalized rewards such as extra course credit, likely meant that the students’ motivation to play decreased significantly (Deci, 1996, p. 25). It is also possible that the collaborative theme of Eco was less engaging to our sample than a more popularized, competitive and traditional zero-sum game design revolving around sabotaging and beating other players (Fennewald and Kievit-Kylar, 2013) – an aspect of games that is traditionally enjoyed especially by males (Chou and Tsai, 2004). In addition to its lacking integration into the students’ planned curriculum, Eco also does not explicitly emphasize gameplay factors that are important to female players such social interaction or tailored emotional experiences (Hartmann and Klimmt, 2006; Schell, 2008). Combined with the fact that females normatively play less than males (Hartmann and Klimmt, 2006), this might at least partially explain the absence of female respondents in the post-gameplay interviews. Despite the small sample size used in our study, however, the amount of information provided by them was rich in detail and featured a sense of coherence in regard to some central gameplay aspects – supporting the notion that even small samples can give interesting results (Wainwright, 1997; Crouch and McKenzie, 2006).

## Limitations

Although the results of our study show some promise, it is also important to acknowledge its limitations. Firstly, all of our respondents were male – it is therefore important that future studies attempt to include more female players so as to avoid skewed research results due to gender differences. Secondly, the version of Eco that was utilized in this study was an unfinished beta version. Future researchers are encouraged to use the finished version of Eco, to avoid some of the issues encountered by our informants (unintuitive game controls, missing in-game textures and items and other related issues). Lastly, if used in a classroom setting, it would appear that integrating Eco as a core element of the curriculum rather than allowing the students to haphazardly play the game on their own leisure would increase the likelihood that the students will interact with the game. This strategy would also allow the teacher and the researcher to form a moderating team where they can engage the students in environment-themed debates and discussions and monitor the students’ progress.

## Data Availability Statement

All datasets generated for this study are included in the article/supplementary material.

## Ethics Statement

The studies involving human participants were reviewed and approved by NSD – Norsk senter for forskningsdata. The patients/participants provided their written informed consent to participate in this study.

## Author Contributions

Both authors participated in generating the initial research idea, conducting the recruitment process, and read and approved the manuscript. KF was responsible for the manuscript writing.

## Conflict of Interest

KF paid for a classroom package of Eco directly from the developer’s website.

The remaining author declares that the research was conducted in the absence of any commercial or financial relationships that could be construed as a potential conflict of interest.
